# A within-subjects trial to test the equivalence of online and paper outcome measures: the Roland Morris Disability Questionnaire

**DOI:** 10.1186/1471-2474-11-113

**Published:** 2010-06-08

**Authors:** Felicity L Bishop, Graham Lewis, Scott Harris, Naomi McKay, Philippa Prentice, Haymo Thiel, George T Lewith

**Affiliations:** 1 Complementary Medicine Research Unit, School of Medicine, University of Southampton, Southampton, UK; 2 School of Medicine, University of Southampton, Southampton, UK; 3 Anglo-European College of Chiropractic, Bournemouth, UK

## Abstract

**Background:**

Augmenting validated paper versions of existing outcome measures with an equivalent online version may offer substantial research advantages (cost, rapidity and reliability). However, equivalence of online and paper questionnaires cannot be assumed, nor can acceptability to respondents. The aim was to test whether online and written versions of the Roland Morris Disability Questionnaire (RMDQ), a standard measure of functional disability in back pain, are equivalent at both group and individual levels to establish whether they can be used interchangeably.

**Methods:**

This is a within-participants equivalence study. 167 participants with back pain fully completed both the paper and online versions of the RMDQ in random order. Participants were recruited from a chiropractic clinic and patient support groups in Southern England. Limits of equivalence were pre-defined as 0.5 RMDQ points, the Bland-Altman range was calculated, and participants' comments were examined using content analysis.

**Results:**

The mean score difference was 0.03 (SD = 1.43), with the 95% Confidence Interval falling entirely within our limits of equivalence (-0.19 to 0.25). The Bland-Altman range was -2.77 to 2.83 RMDQ points. Participants identified unique advantages and disadvantages associated with each version of the RMDQ.

**Conclusions:**

The group and individual level data suggest that online and paper versions of the RMDQ are equivalent and can be used interchangeably. The Bland-Altman range appears to reflect the known measurement properties of the RMDQ. Furthermore, participants' comments confirmed the potential value to be had from offering them the choice of completing the RMDQ online or on paper.

## Background

Use of the Internet for health care research has grown in the last fifteen years [1,2]. The internet provides an alternative to postal means of administering self-completed outcome measures. Purported research advantages include improved data input accuracy (no manual data entry, immediate checks for incomplete responses, additional 'meta'-data such as duration/date of response can be logged), reduced costs (no manual data entry, no printing or postage costs), reduced response time, increased response rates, and increased ease of administration [3-7]. There are also potential benefits to participants: online questionnaires can be completed wherever the participant has access to the Internet (work, home, holiday) and there is no need to return them by post.

Trials collecting data using online questionnaires only would however exclude all those people who are not Internet users, which in 2007 was approximately 35% of the UK population [8]. There is also the potential for higher drop-out rates due to the less personal nature of online studies and a subsequent feeling of having less involvement in the research [1,9,10]. Some patients may have conditions that make it difficult, painful, or even impossible to use a computer. Researchers could offer participants the choice to complete either online or paper versions of outcome measures. However, it is not safe to assume that online versions of pre-existing outcome measures will be equivalent to written versions [1,2]. Outcome measures may have different psychometric properties when transferred online and may obtain systematically different responses compared to paper versions. Researchers recommend first testing the online method against its written counterpart and some have documented systematic differences between online and written versions of questionnaires [1] while others demonstrate equivalence [4,6,11].

Back pain is a significant epidemiological and economic problem in the UK and further research is needed to establish the most appropriate, cost effective treatments for back pain [12]. Such studies require the use of responsive outcome measures in order to increase the likelihood that true treatment effects will be detected [13]. The Roland Morris Disability Questionnaire (RMDQ) is one of the most well-validated back-pain specific measures of functional status and is widely used as an outcome measure in clinical trials [15,16]. It has been recommended for use as an International Standard questionnaire in back pain research and was used as a main outcome measure in the United Kingdom back pain exercise and manipulation (UK BEAM) trial [15,17,18]. We could not identify any studies evaluating the equivalence of online and paper versions of the RMDQ. Therefore, we aimed to develop an online version of the RMDQ and to test its equivalence against the paper version. In doing so, we hoped to provide an evidence-base for future trials concerning patient-centred, efficient procedures for measuring outcomes. We hypothesised that the two versions would be equivalent, and our objectives were to assess:

1. The equivalence of total scores between online and paper versions of the RMDQ,

2. The equivalence of missing data rates, and

3. Participants' perceptions of completing each version.

## Methods

### Design

We used a within-subjects equivalence design, in which all participants completed both versions of the RMDQ on the same day.

### Participants and Procedure

Participants were recruited between January 2008 and April 2009 from a local chiropractic clinic and patient support groups (we advertised the study to people with back pain through BackCare and the National Ankylosing Spondylitis Society, both of which provide information and support to people with back pain). Eligible individuals had to have back pain (scoring ≥1 on the RMDQ), be able to access the Internet, and be at least 18 years old. After giving informed consent, participants were given a study pack containing a participant number and instructions as to which RMDQ to complete first, a paper RMDQ, instructions for completing the online RMDQ, and an additional question sheet. Order of completion was randomised using participant number -- before putting together the study packs, the complete list of participant numbers was randomised to receive instructions to complete either the online or the paper RMDQ first. This randomisation was balanced --for each group of 10 participant numbers, five were randomised to complete the online RMDQ first and five were randomised to complete the paper RMDQ first. Participants were instructed to complete both versions of the questionnaire on the same day to ensure the same underlying back pain disability when completing each version (the RMDQ asks respondents to 'think about yourself today' when answering). A laptop computer was available at the clinic for participants to use to complete the online version (but participants were free to complete either one or both versions elsewhere if they wanted to).

### Questionnaire Measures

#### The RMDQ

We used the original 24-item paper version of the RMDQ [13] with the response options (Yes/No instead of just Yes) used by Patrick et al. [14]. The online version was developed using online questionnaire software (Psychosurvey) provided by the School of Psychology, University of Southampton. It was designed to mimic the format of the paper version: answers were given by selecting either a 'yes' or 'no' radio button and all questions were displayed on one page. The instructions were repeated after question 12 so they remained visible throughout. For both versions, answering Yes to a question scores 1, answering no scores 0. Items are summed to give a total score (range 0 to 24), where 24 represents the maximum disability score.

#### Additional Questions

We collected standard socio-demographic data and information about the history of participants' back pain. Participants provided written responses to four open-ended questions concerning their perceptions of the advantages and disadvantages of each version of the RMDQ.

### Data Analyses

Online RMDQ scores were automatically entered into an Excel file and then transferred into SPSS (Version 16.0). Data from the paper questionnaires were entered manually. The equivalence of online and paper RMDQs was tested regarding (1) mean total score difference (2) within-subject score differences and (3) differences in responses to individual questions. These analyses thus took into account (1) individual respondents' score differences and (2) differences in responses to individual questions. Even if mean scores on two versions of an outcome measure are identical, individual respondents may have large score differences and/or there could be differences on individual items.

#### Analysis of mean score difference

Difference scores were calculated for each participant (difference d = paper RMDQ total score - online RMDQ total score), and shown graphically to approximately follow a Normal distribution. A paired t-test was used to calculate the mean score difference and the associated 95% confidence interval. An Analysis of Variance (ANOVA) model was used with treatment, period and patient effects to account for any differences due to the order of completing the online and paper RMDQs.

To test the hypothesis that the RMDQs were equivalent, a limit of equivalence was defined, i.e. a value for the mean score difference that needed to be exceeded to determine that the two RMDQs were not equivalent. The limit of equivalence is a somewhat arbitrary value that we set at 0.5, to provide a strict test of the equivalence of the two RMDQs: a mean score difference of up to 0.5 cannot be rounded up to a whole RMDQ point. If the entire 95% confidence interval for the mean difference falls between -0.5 and +0.5 then we can be confident that the true difference was no greater than 0.5 in either direction, which is strong evidence of equivalence given the scoring system of the RMDQ. Our sample size calculation showed that 156 participants were required to have 80% power to detect (at a 5% significance level) a mean score difference of 0.1 (assuming standard deviation = 2).

#### Exploratory analysis of individuals' score differences

The Bland and Altman range (illustrated on Bland-Altman plots) was used to indicate the extent to which scores on the online RMDQ are likely to differ from scores on the paper RMDQ for individual participants [19]. This range is the difference between the lower and upper limits of agreement, where limit of agreement = mean difference ± two standard deviations of the difference. In the Bland and Altman plot, individual participants' score differences were plotted against their mean score, illustrating individual score differences and the effect of the mean score on the score difference.

#### Sensitivity Analysis

We repeated our analyses of mean and individual score differences excluding participants scoring less than 5 on either or both versions of the RMDQ (i.e. participants with scores falling within the typical measurement error of the RMDQ [20]).

### Analysis of responses to open-ended questions

Responses to the open-ended questions were entered into MS Word and subjected to a simple content analysis [21] in which all individual statements were categorised as being positive or negative comments about the online RMDQ or the paper RMDQ, or neutral comments. Similar comments were grouped together inductively within these overarching categories.

## Results

### Participants

Questionnaires were received from 183 participants. Sixteen were excluded (4 did not have back pain, 11 only completed one version of the RMDQ and 1 answered less than 50% of questions on one version), giving a final sample of 167. Order of completion could not be confirmed for 8 participants and so for the adjusted ANOVA that includes a period effect the sample size is further reduced to 159. Due to an administrative oversight some background data were not collected for 52 participants. The characteristics of participants are shown in Table 1. Participants who completed the online RMDQ before the paper version had similar characteristics to those who completed the paper version first.

**Table 1:** Clinical and Demographic Characteristics of Participants.

	Whole sample (n = 167)	Online then paper (n = 76)^a^	Paper then online (n = 83)
Gender			

Male	60 (35.9%)	23 (30.3%)	35 (42.2%)

Female	90 (53.9%)	43 (56.6%)	42 (50.6%)

Missing data	17 (10.2%)	10 (13.2%)	6 (7.2%)

Age			

Mean Range	46.28 18 to 78	44.79 19 to 74	46.66 18 to 78

Missing data (n, %)	48 (28.7%)	19 (25%)	27 (32.5%)

Duration of back pain (yrs)			

Mean Range	9.25 0.02 to 50	8.68 0.04 to 40	9.36 0.02 to 37

Missing data (n, %)	49 (29.3%)	20 (26.3%)	27 (32.5%)

Employment status			

Unemployed	1 (0.6%)	1 (1.3%)	0 (0.0%)

Retired	24 (14.4%)	10 (13.2%)	12 (14.5%)

Employed	66 (39.5%)	30 (39.5%)	32 (38.6%)

In education	16 (9.6%)	9 (11.8%)	7 (8.4%)

Unable to work due to back pain	1 (0.6%)	0 (0.0%)	1 (1.2%)

Other	7 (4.2%)	3 (3.9%)	4 (4.8%)

Missing data (n, %)	52 (31.1%)	23 (30.3%)	27 (32.5%)

^a ^8 participants did not confirm the order in which they completed the questionnaires and so are excluded from order of completion analyses.

### Missing Data

There were 15 missing responses on the online RMDQ (equivalent to .004% of items) and 3 on the paper RMDQ (.0007%); they were not concentrated on any one item. Cases with missing data were excluded from the following analyses.

### Mean Score Differences

The mean score difference between the paper and online RMDQ was 0.03 (SD = 1.43). The 95% confidence interval around this mean difference ranged from -0.19 to 0.25. This falls within our predefined acceptable limit of equivalence (+/- 0.5). The analysis adjusting for a difference in period also falls well within the preset limits of equivalence (Table 2). Thus the mean total scores on the paper and online RMDQ are equivalent.

**Table 2:** Mean (SD) scores on the RMDQs, mean difference, adjusted mean difference and 95% range of agreement.

	Online RMDQ	Paper RMDQ	Mean Difference (95% CI)	Adjusted Mean Difference (95% CI)	Bland Altman 95% range of agreement
Full dataset	7.52 (5.43)	7.55 (5.21)	0.03 (-0.19 to 0.25)	-0.04 (-0.26 to 0.18)	-2.77 to 2.83

Online first (n = 76)	7.34 (5.14)	6.97 (5.24)			

Paper first (n = 83)	7.46 (5.72)	7.75 (5.06)			

### Individual-level Score Differences

**Figure 1:**
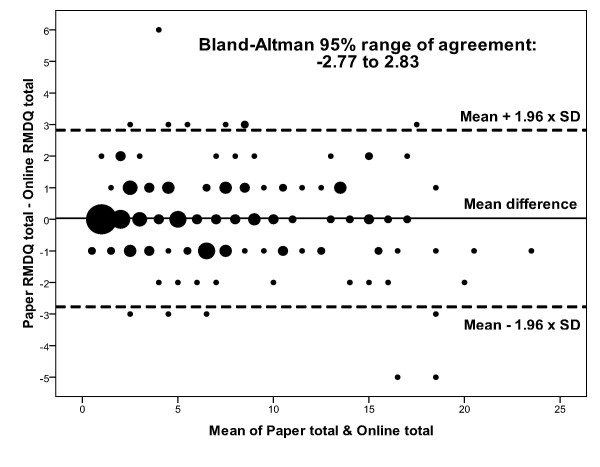
**Bland-Altman plot of difference scores against average scores for online and paper RMDQs**.

The Bland-Altman graph plots individual participants' difference scores against their mean total scores (Figure 1). The Bland-Altman range is -2.77 to 2.83, and the plot shows that there is no obvious relationship between the score difference and the mean score. The intra-class correlation coefficient (2-way mixed model for absolute agreement) for online and paper RMDQs is 0.965 (95% CI: 0.953 to 0.974) which shows a very good inter method reliability.

### Sensitivity Analysis

Excluding all cases with scores below 5 on either RMDQ did not meaningfully alter our findings. The mean score difference increased slightly (0.11, SD = 1.41) but the 95% confidence interval remained acceptable (-0.17 to 0.38); the Bland-Altman range shifted slightly to -2.65 to 2.86.

### Participants' Comments

Typical examples of comments are shown in Table 3. Participants made slightly more positive comments about the online RMDQ than about the paper RMDQ (61 versus 53). They most commonly reported that the online RMDQ was easy (17 comments) and quick (19 comments) to complete. However, participants also found the paper version easy (16 comments) and quick to complete (9 comments). Some unique positive features of the online RMDQ were reported, namely being able to change answers easily, finding it easier to submit responses (at the press of a button rather than via a post box), and seeing it as more environmentally friendly than the paper version. Unique positive features of the paper RMDQ included being able to see the whole questionnaire at once, and being more familiar with the modality. Both versions were described as physically painful to complete by small numbers of different participants who, for example, found it painful to walk to a post box or to sit at a desk and use a computer. Participants made more negative comments about the online RMDQ than they did about the paper RMDQ (34 versus 17). Negative features unique to the online RMDQ were predominantly related to difficulties using the laptop (15 comments), but also included disliking the layout (5 comments), finding it impersonal (2 comments) or generally disliking computers (3 comments), and finding it painful to use a computer (1 comment). Negative features of the paper version included: seeing it as not environmentally friendly (3 comments), finding it physically painful to complete (2 comments) or post back (1 comment), having to turn the page over (3 comments), finding it difficult to change answers (1 comment). A further 13 comments expressed neutrality concerning the different versions of the RMDQ.

**Table 3:** Typical comments from participants.

	Online RMDQ	Paper RMDQ
Positive features	"Much easier to change an answer after further consideration if necessary" "Saves paper" "Saves walking to mailbox which can be a problem due to back pain."	"Knew what to expect" "Can see full overview of questions"

Negative features	"Sometimes navigating with the mouse was not always easy, i.e. clicking in the yes/no boxes" "Not keen on using computers" "I am not a great fan of working online"	"Difficult to change things if you filled in the wrong box" "Not environmentally friendly"

## Discussion

Our group-level data suggests that mean group scores on the online and paper versions of the RMDQ are equivalent. The 95% Confidence Intervals for the raw mean score and the adjusted mean score difference were both well within our pre-defined limits of equivalence. While there were slightly more missing data points on the online RMDQ than the paper RMDQ the proportion of missing data was very low for both versions, and it would be possible to programme future online versions of the RMDQ to prevent people submitting a questionnaire containing missing data (although such an approach might result in fewer respondents overall). According to our content analysis of responses to open-ended questions, participants were able to identify unique positive and negative features of each version of the RMDQ. They also, in similar numbers, found each version easier and quicker than the other, and a very small number reported finding it physically painful to complete one version or the other. These findings support the value of offering research participants a choice of modality in completing outcome measures. Doing so might enhance patients' experiences of taking part in research and reduce the burden of participation.

Our Bland-Altman analysis suggested that in 95% of individuals who complete the two versions of the RMDQ, total score differences can be expected to be as great as approximately plus or minus three RMDQ points. While this appears to be a substantial difference, it is consistent with the known measurement properties of the RMDQ. The minimum level of detectable change for the RMDQ (i.e. the observable change deemed necessary to be distinguishable from measurement error) is 5 points [20]. The Bland-Altman range identified in our study is thus likely to represent this inherent level of variability (measurement error) in the RMDQ rather than any additional systematic difference between online and paper versions. Equivalence between online and paper versions of established questionnaires is emerging as a common finding in the literature [22] and our results are consistent with this trend.

Our methodology is limited in some respects. Our findings could be an artefact of our crossover design; participants could have remembered their responses to the first version when completing the second version of the RMDQ. If this occurred then it would have contributed to the consistency we found across online and paper versions. We would recommend future cross-over studies recruit from a known stable population to enable a longer gap between the completion of online and paper questionnaires. Our crossover design enabled us to investigate individual-level equivalence. However, a parallel groups design (randomising participants to complete either the written or the Internet version) would offer a valuable complement, in that using a large, representative sample would control for both known and unknown confounders and eliminate the possible bias due to memory. We recommend that such a study is undertaken to strengthen the evidence base in this area. Further limitations that we acknowledge here and which should be overcome in future research include our lack of control over or monitoring of the time gap between completions of the two versions of the questionnaire, and our lack of independent verification of the order of completion of the two versions of the questionnaire.

## Conclusions

This study provides initial evidence that an online version of the RMDQ is equivalent to its paper counterpart, and research participants would appear to value being given the choice of which version to complete. Therefore it should be possible to use the two versions interchangeably to benefit from the research advantages of online administration and to enhance participants' experiences of research. A large scale randomised study is needed to confirm these preliminary conclusions.

## Competing interests

The authors declare that they have no competing interests.

## Authors' contributions

FLB, GL, and GTL conceived of the study. FLB, GL, GTL, NM, PP, and HT participated in its design and coordination. FLB and SH performed the statistical analysis. All authors helped draft the manuscript and read and approved the final version.

## Pre-publication history

The pre-publication history for this paper can be accessed here:

http://www.biomedcentral.com/1471-2474/11/113/prepub
